# Inverse heat mimicking of given objects

**DOI:** 10.1038/srep43288

**Published:** 2017-03-02

**Authors:** Ahmed Alwakil, Myriam Zerrad, Michel Bellieud, Claude Amra

**Affiliations:** 1 Aix Marseille Univ, CNRS, Centrale Marseille, Institut Fresnel, Marseille, France; 2 Université de Montpellier2, LMGC, Montpellier, France

## Abstract

We address a general inverse mimicking problem in heat conduction. The objects to cloak and mimic are chosen beforehand; these objects identify a specific set of space transformations. The shapes that can be mimicked are derived from the conductivity matrices. Numerical calculation confirms all of the analytical predictions. The technique provides key advantages for applications and can be extended to the field of waves.

The field of transformation optics has been extensively used in recent years in a variety of fields, including optics and microwaves, acoustics and mechanics, and hydrodynamics and seismicity^1^,^2^,^3^,^4^,^5^,^6^,^7^,^8^,^9^,^10^,^11^,^12^,^13^,^14^,^15^,^16^,^17^. Within this framework, a great number of papers can be found that describe new phenomena, such as invisibility and mimicking, together with their related devices, such as cloaks, concentrators and rotators, and absorbers. One basic idea lies in the gradient optimization of the physical parameters, which allow for matching of the admittances at the frontier of the overcoats that surround the objects to cloak or mimic, and this optimization is given by the space transformation. Metamaterials are then required to satisfy the gradient constraints, and they are approached by micro-structures of isotropic materials supported on homogenization techniques.

Following these works devoted to waves, analogies have been emphasized between optical propagation and thermal diffusion, including multilayers and micro-cavities, the diffraction process, and cloaking. Such analogies have promoted the extension of the transformation techniques to the field of thermal phenomena^18^,^19^,^20^,^21^,^22^,^23^,^24^,^25^,^26^,^27^,^28^,^29^,^30^,^31^,^32^,^33^, at least for conduction. Numerical results have been given to confirm the predictions and concerned the isotherms correction and their quantification at the cloak exit of different devices for invisibility, concentration and rotation^26^. In this paper, we first extend this previous work and generalize the mimicking problem to the thermal field to produce thermal illusions^34^,^35^,^36^,^37^,^38^,^39^,^40^.

However, in most problems of transformation optics, the objects to cloak or mimic emphasize arbitrary matrix gradients of their physical parameters, thereby making them difficult to produce. Furthermore, this constraint reduces the field of practical applications because we do not really choose what we are able to hide or camouflage. In practice, one would be interested in working with predefined objects of given shape and conductivity, and these objects would be over-coated to mimic other predefined objects beyond the cloak. Such an inverse engineering problem introduces constraints that restrict the range of space transformations, thereby classifying the set of objects that can be mimicked with another one which is beforehand chosen and must be over-coated. Because all conductivities are forced, the remaining degrees of freedom lie in the shape of the objects and in the set of specific cloaks that surround them.

To address this topic, we start with a general analysis of the inverse problem. This analysis involves a 2D polar geometry allowing for analytical calculation. Next, the specific transformations are emphasized, the shapes are identified, and the cloaks are designed. The last part is devoted to numerical validation before the conclusion is presented.

## Inverse Transformation

**Figure 1:**
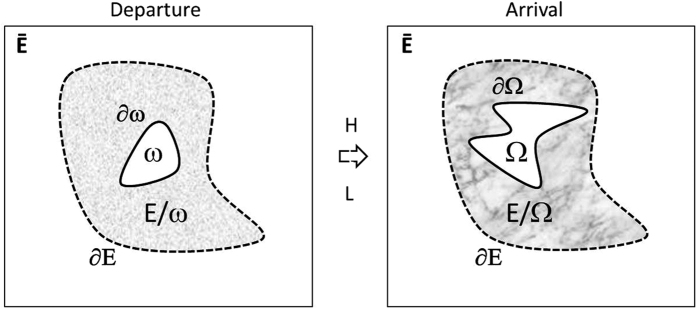
On the right side (arrival space), Ω is the object to overcoat with a cloak limited by surfaces ∂E and ∂Ω. The function of the cloak is to mimic in Ē the flux of another predefined object ω given on the left side (departure space). H and L are space transformations from the departure space to the arrival space (see text).

Consider in Fig. 1 (right side) a thermal object with conductivity matrix k_Ω_ within domain Ω limited by surface ∂Ω. This object scatters a specific heat flow **q**_Ω_ in its surroundings in relation to its geometry ∂Ω and its thermal parameters, together with the position and nature of thermal sources. Assume now that one aims at mimicking the heat flux **q**_ω_ of another thermal object shown in Fig. 1 (left side), with conductivity k_ω_ within domain ω limited by surface ∂ω. To achieve this goal, a cloak is used to fill domain E\Ω limited by surfaces ∂E and ∂Ω. This cloak surrounds the thermal object Ω, and its function is to transform its flux **q**_Ω_ to mimic outside the cloak the flux **q**_ω_ of object ω. In other words, one should have in region Ē above the cloak (above ∂E):


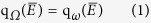


Following Fig. 1, one key problem is to find the cloak filling region E\Ω and its thermal parameters. The nature and existence of this cloak depend on the two objects to be cloaked (Ω) and mimicked (ω), including both their thermal parameters and their frontiers. In addition, the cloak depends on the ω surrounding (E\ω). To solve this problem, we use transformation optics^1^,^2^,^3^,^4^,^5^, a well-known technique that first consists of:


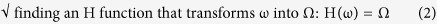



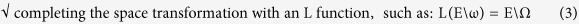


As a result of continuity, these functions satisfy:


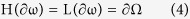



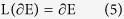


Therefore, one can find an infinity of cloaks to satisfy **q**_Ω_ = **q**_ω_ in domain Ē. Indeed, the external shape ∂E of the cloak surface is arbitrary, and for each shape, there is a large choice of transformations.

In fact, in this inverse problem, difficulties appear from the constraints that accompany the transformations. Indeed, in the engineering problem we address here, the two objects are predefined with given thermal parameters and frontiers, whereas transformation optics^1^,^2^,^3^,^4^,^5^, which provide the ideal solution of this mimicking problem, force several relationships between the two objects (ω and Ω). First, the thermal parameters of the objects should be connected as:


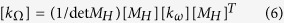



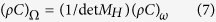


where (ρC) is the product of bulk density and heat capacitance, and [M_H_] is a matrix that we further detail; [M_H_]^T^ is its transposed form, and detM_H_ is its determinant.

Similar constraints must be satisfied for the cloak, that is:


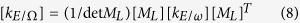



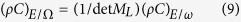


At this step, the degrees of freedom must be analyzed. Let us first consider relation (6) related to the anisotropic conductivities of the objects to cloak (k_Ω_) and to mimic (k_ω_). When these two conductivity matrices are fixed, relation (6) reduces the set of possible H transformations with a constraint in the form:


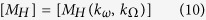


Hence, solutions of (10) will emphasize a specific set {H_μ_} of transformations that connect the two object conductivity matrices in ω and Ω. At this step, the remaining degrees of freedom lie in the frontiers (∂ω and ∂Ω), that is, in the shape of the objects. Therefore, if we choose one object Ω to cloak (conductivity and shape are predefined for Ω), the class of objects ω (with given conductivity) that can be mimicked exhibits a set of shapes belonging to:


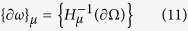


Notice that shape and conductivity can be reversed in this inverse problem. One interesting point is that the cloak introduces no additional constraint on the object to coat. Indeed, when all frontiers (∂ω, ∂Ω and ∂Ε) are given, the second L transformation can always be determined, and relation (8) gives the matrix conductivity of the cloak. In the last step, the cloak can be approached with homogenization techniques.

However, one cannot ignore further constraints resulting from the (ρC) products given in relations (7, 9) that force the matrix determinants of M_H_ and M_L_. When these products are forced, the additional constraints again restrict the set of transformations and may prevent the existence of solutions. Otherwise, one must consider that the (ρc) products are arbitrary, at least for one of the two objects. Another approach to overcome this issue is to start with a problem first limited to the static regime of the heat equation and focus the investigation on the connection between shape and conductivity. Subsequently, the (ρc) constraints will be analyzed case by case for the dynamic regime.

## Results

### Analytical formulation of the 2D problem in polar coordinates

Here, we focus on relation (6) and search for the H transformations that can satisfy it when the two conductivity matrices are forced. For this purpose, the M_H_ matrix is first analyzed in detail. An analytical calculation can be developed if we simplify the problem by considering a 2D geometry with polar coordinates (r,θ). The transformation is written as:


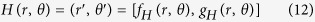


where r and r’ are the radius vectors in the departure and arrival spaces, respectively, and θ and θ’ are the polar angles in the departure and arrival spaces, respectively. The M matrix is similar to that which is classically obtained with a Jacobian. Here, it is introduced to express the same physical gradient in different polar coordinate systems, that is:


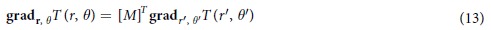


with the temperature T and


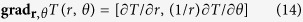



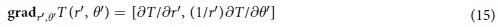


We obtain a matrix with dimensionless coefficients, that is:


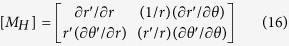


### Case of a heterogeneous isotropic medium (ω) to mimic

Let us here consider that the ω object to mimic is isotropic (but still heterogeneous). The result is that k_ω_ is a scalar quantity, and this allows us to turn relation (6) into:


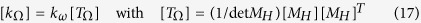


with [T_Ω_] a symmetric tensor with unity determinant, so that:


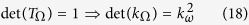


At this step, the constraint on the H transformation is fully given by this tensor, which is now forced to follow:


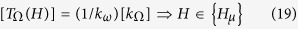


Because the two conductivities are given, this condition reduces the range of shapes ∂ω that can be mimicked with a material of frontier ∂Ω surrounded by a cloak (see relation (11)).

Finally, introducing (16) into (17) gives the tensor T_Ω_ in terms of two vectors **u** and **v** as follows:


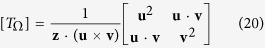


where (.) and (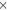) are the scalar and vector products, respectively, and:


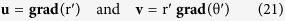


In summary, the conductivity matrix [k_Ω_] of the object to cloak is entirely given in relation (20) by the knowledge of two vectors 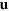 and 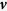 resulting from the H transformation. Writing the conductivity as:


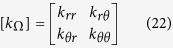


we obtain its matrix coefficients as:


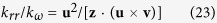



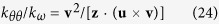



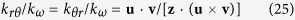


with (**u**, **v**, **z**) a direct trihedron. These relations (23–25) are the conditions to satisfy in the inverse transformation problem. Since all conductivities are chosen beforehand, the unknowns are the two vectors (**u**,**v**) that are transformation-related. In accordance with (18), they also yield:


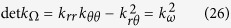


We notice that relation (25) requires the conductivity matrix to be symmetrical, which is not a constraint. In addition, we keep in mind that in the dynamic (temporal) regime, one would also have to consider the transformation of the (ρc) products given as:


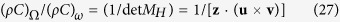


### Case of a diagonal conductivity matrix to cloak

Until now, we assumed that the object ω to mimic was isotropic (but still heterogeneous). In addition, we here consider that the anisotropic object Ω to cloak has a diagonal conductivity matrix, that is:


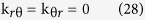


Following (26), the two other conductivity coefficients in the matrix follow:


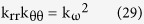


Furthermore, relation (25) forces the orthogonality of the two real **u**, **v** vectors that define the [T_Ω_] tensor, so that the difference ϕ_v_-ϕ_u_ in their directions follows:


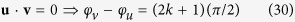


with k a relative integer. To avoid negative values of the ρC products (see eq. (27)), we restrain this direction difference to the condition:


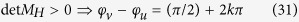


This leads to the result:


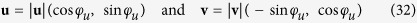


In addition, this yields the condition:


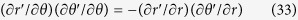


Among this set of solutions that allow the conductivity matrix [k_Ω_] to be diagonal, one can explore a subset with the direction property:


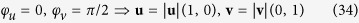


Following (21), the final result is:


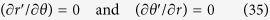


so that the polar and radial variations are independent in the transformation, that is:


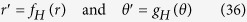


With this last property, the conductivity matrix becomes:


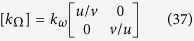


with:


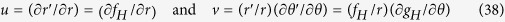


As a consequence, the key equations of the inverse problem can now be summarized as:


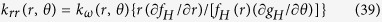



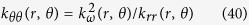


To conclude, relations (39–40) guarantee the fact that the object Ω with diagonal conductivity matrix [k_Ω_(k_rr_,k_θθ_)] can be cloaked to mimic (in the static regime) a class of isotropic objects ω with heterogeneous isotropic conductivity k_ω_. The shape ∂ω of these objects can now be discussed because the f_H_ and g_H_ functions are given versus the conductivity values.

In addition, we notice again that in the dynamic regime, the additional constraint on the (ρc) product is written as:


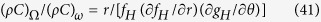


In some situations, these constraints can be compatible with those of the conductivities, for which reason they are recalled at each step.

### Identification of the shapes that can be mimicked

Next, we work with relation (39), which identifies the H transformation because the 2 conductivities [k_Ω_] and k_ω_ are fixed beforehand. When this transformation is known, we determine the shapes ∂ω that can be mimicked when ∂Ω is chosen, on the basis of (11,19). The key equation to satisfy is rewritten as:


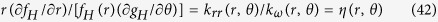


where the conductivity ratio η(r,θ) is fixed. The unknowns are the f_H_ and g_H_ functions.

#### Case of similar heterogeneities in both objects

We first assume the heterogeneities to be related in both objects, in the sense that the radial conductivity values k_rr_ in Ω are proportional to those of k_ω_ in ω, that is:


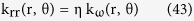


with η a constant. From (42), we obtain with μ another constant:


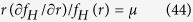



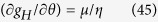


which yields the transformation:


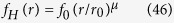



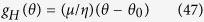


with the constants f_0_, r_0_ and θ_0_, and |μ/η| ≤ 1. Such transformations are rather simple since they are a combination of a μ^th^ power of the radius with a scaled rotation. At this step, the f_H_ and g_H_ functions are known and can be reversed as:


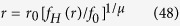



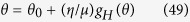


so that every shape ∂Ω given by r′ = f_H_(r) and θ′ = g_H_(θ) gives rise to the range of shapes {∂ω(η,μ,f_0_,r_0_,θ_0_)} that can be mimicked.

**Figure 2:**
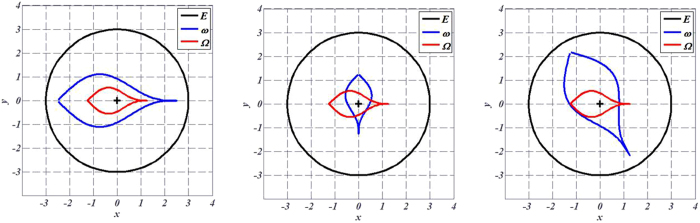
Examples of different ω objects (with blue frontiers) that can be mimicked when the same Ω object (with red frontier) is cloaked. The 2 conductivities are chosen beforehand (with k_rr_ proportional to k_ω_ - see text), which forces the nature of the transformation (see text). The external frontier of the cloak is circular and plotted in black.

Shape illustrations are given in Fig. 2 below for an arbitrary geometry of the object Ω to cloak. The ∂Ω frontier is plotted as a red line, whereas the cloak is circular with an external frontier ∂E plotted in black. These arbitrary frontiers ∂E and ∂Ω are the same for all drafts in Fig. 2. Alternatively, the frontiers ∂ω of the objects ω that can be mimicked are plotted as a blue line and result from the inverse H transformation given in (48–49). As seen in Fig. 2, the objects can be widened (left figure) or reduced, rotated (middle figure), or both (right figure), depending on the set of parameters. Notice that, until now, we did not discuss the cloaks that will allow the thermal illusion (the mimicking effect).

Now that such ∂ω surfaces and H transformations have been determined from the knowledge of η = k_rr_(r,θ)/k_ω_(r,θ), we also have to take into account the anisotropic nature of the Ω conductivity. This leads us to analyze the second (polar) conductivity coefficient k_θθ_. In fact, (40) and (42) give:


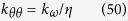


so that the object Ω to cloak must be diagonal in the form:


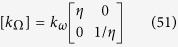


It is also interesting to analyze under which conditions this Ω object can be isotropic. In fact, from relation (42), the value η = 1 leads to k_rr_ = k_θθ_ = k_ω_. This last situation indicates that, if the objects to cloak and to mimic have the same heterogeneous isotropic conductivities, then the range of shapes {∂ω_μ_} that can be mimicked follows:


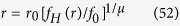



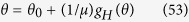


with |μ| ≤ 1. With regard to (48–49), we notice that only the polar variation is modified. This range of surfaces characterizes the transformations that hold the scalar (isotropic) conductivity invariant (k_ω_ = k^Ω^). In other words, from (17), they follow the tensor condition:


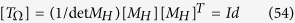


where Id is the identity matrix. Hence, when the radial transformation is not linear (μ ≠ 1), a scaled rotation in the form (θ′ = μθ) is necessary to hold the scalar conductivity.

As previously discussed, we also must consider the (ρc) constraints involved in a transient or dynamic regime, to determine whether compatibility can be found with those of conductivity. With η = 1, these products are related as:


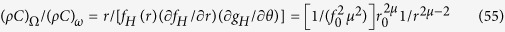


Such a relationship could be used to choose the μ value to fit the (ρc) ratio of the objects in the case where this ratio behaves as a n^th^ power of r, with n = 2(1-μ) ≥ 0. In this last situation, all constraints on thermal parameters would be satisfied, allowing for generalization of the previous techniques to the transient regime. However, in practice, the optimal situation is that of a constant (ρc) ratio given by μ = 1, that is:


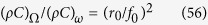


Following (52–53) the associated transformation is given by:


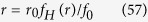



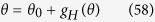


These simplified relations (57–58) are the simplest ones that allow the mimicking technique in the transient regime for given isotropic objects. The transformation is reduced to a simple combination of a rotation and a homothety.

To conclude this sub-section, it should be noted that invisibility is usually discussed in the case of the same constant conductivity for ω and its surrounding (E\ω), that is k_ω_ = k_E\ω_, which allows for mimicking the homogeneous region. The result is that the field perturbation (scattering or diffraction) resulting from the Ω object vanishes at the external frontier ∂E of its cloak, as if this cloak were acting as a perfect antireflective device, regardless of the excitation conditions. Single H transformations have been widely used in this situation with the range of parameters: μ = 1, f_0_ = 1, θ_0_ = 0, that is, f(r) = r/r_0_ and g(θ) = θ. Furthermore, most often, a circular geometry is considered with a central disk transformed into another one. For all these geometries, the scalar isotropic conductivity is the same in ω and Ω, whereas the ρc product follows (ρc)_Ω_ = (ρc)_ω_ r_0_^2^. Vanishing values of r_0_ decrease the ρc product in Ω. In all situations, the cloak conductivity is anisotropic and heterogeneous (see further).

#### Two other specific cases (radial or polar η function)

Until now, the conductivity ratio η was assumed to be constant. Here, we consider the situation where this ratio given in (42) depends on a unique radial or polar variable (r or θ). Let us start with the radial property:


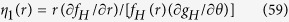


where η_1_ is chosen beforehand. The result is:


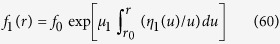



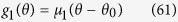


where f_0_, μ_1_ and θ_0_ are constants and |μ_1_| ≤ 1. The conductivity matrix [k_Ω_] is anisotropic and diagonal with:


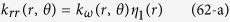



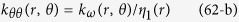


that is:


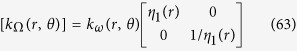


At this step, it should be noted that the inhomogeneity of conductivity modifies the shape of the object that can be mimicked. Indeed, when f_1_(r) and g_1_(θ) are given to fix Ω in the arrival space, ω is rebuilt in the departure space as:


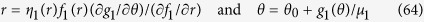


Another specific case is given by a polar dependence of the conductivity ratio:


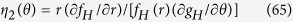


where η_2_ is again forced. The result is:


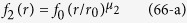



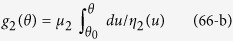


The conductivity matrix is still anisotropic with:


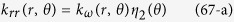



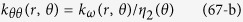


that is:


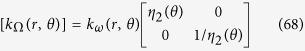


The inhomogeneous conductivity again modifies the shape of the object to mimic in the form:


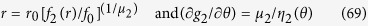


However, in this case, the θ inversion is less immediate.

#### General solution

Eventually, the general solution can be directly found from the previous solutions. Indeed, eq. (42) first forces the conductivity ratio η to be a function with separate variables; this condition is specific to the material to be camouflaged and is a constraint associated to the diagonal property of the conductivity matrix.

We obtain:


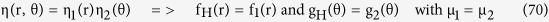


and the anisotropic conductivity matrix:


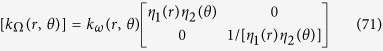


These last relationships broaden the range of transformations that allow the inverse mimicking. To summarize, in this work, the two objects to cloak (Ω) and to mimic (ω) are predefined in conductivity, together with the ∂Ω frontier, which forces the set of possible H transformations and thus the range of ∂ω shapes. If one aims at designing arbitrary thermal parameters of an object Ω and the cloak around it to mimic a given object ω, then the solutions will always exist, regardless of the frontiers ∂ω and ∂Ω.

## Discussion

### Designing the cloaks

Until now, we considered the H transformations that connect the objects to cloak and mimic; however, we did not address the L transformation that gives the cloak E\Ω. The problem of the cloak is different in the sense that there is a large degree of freedom. In fact, the cloak is limited by surface ∂Ω which is given and by surface ∂E that we can arbitrarily choose. Furthermore, there is no need a priori for its conductivity matrix to be diagonal, as was assumed for the Ω object. In the general case, one may address an anisotropic heterogeneous cloak. Following (8), the conductivity matrix of the cloak is known from the L transformation that ensures:


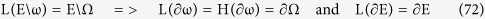


Emphasizing analytical examples is not an easy task when the two cloak frontiers are arbitrary. This is the reason why well-known geometries are most often addressed. However, analytical calculation can still be performed with specific geometries given by star domains, which we now address.

Let us assume that all domains (ω, Ω and E) are star domains and consider that the frontiers ∂ω and ∂E are given in the form r_ω_(θ) and r_E_(θ). Next, one can build a simple “canonical” L function that transforms E\ω to give the cloak E\Ω as:


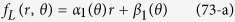



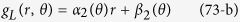


The (α_i_, β_i_) coefficients can be directly calculated to satisfy (72), and this yields:


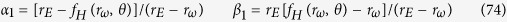



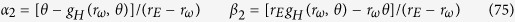


with:


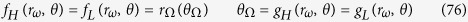


and:


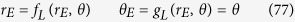


Such an L transformation is homothetic for each polar angle. It is fully determined from the 2 frontier equations (∂ω and ∂E) and from the H transformation. It can be used to calculate the cloak conductivity. For that purpose, we must use the general relation (8) and not that (36) of the diagonal objects because the derivative ∂r’/∂θ is not zero for L.

**Figure 3:**
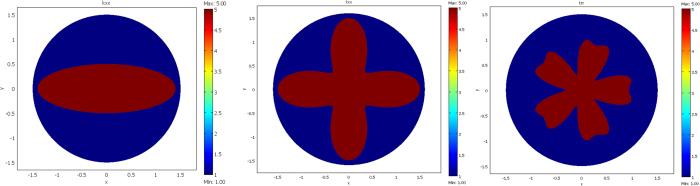
Departure (or virtual) space- Three different shapes of the starting object ∂ω to mimic (from the left to the right): an ellipse, a flower and a butterfly. The color scales are for the conductivity.

**Figure 4:**
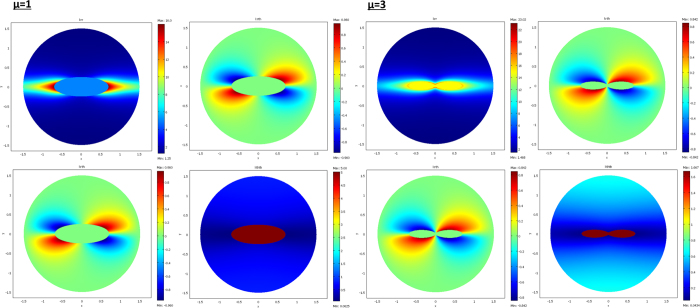
Cloak matrix conductivities to mimic the ellipse of Fig. 3, with parameters μ = 1 (left figure) and μ = 3 (right figure).

**Figure 5:**
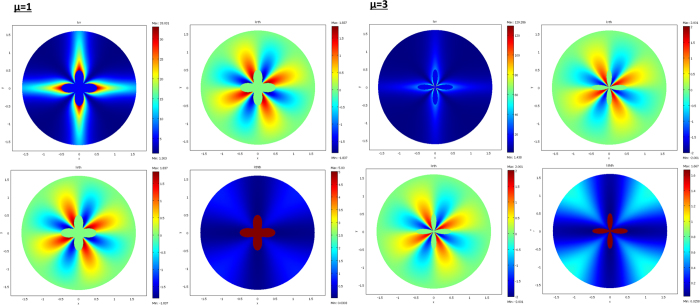
Case of the flower, with μ = 1 (left figure) and μ = 3 (right figure).

**Figure 6:**
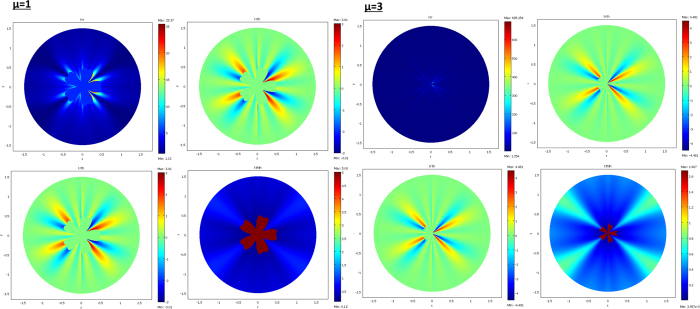
Case of the butterfly, with μ = 1 (left figure) and μ = 3 (right figure).

The results are given below in Fig 3, 4, 5 and 6. The conductivity ratio η is constant, so that the H transformation is given by (46–47). For the sake of simplicity, we first avoid the rotation in the H transformation, which we obtain with μ = η and θ_0_ = 0 (rotation is discussed further). Hence, the H transformation is given by:


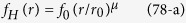



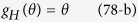


and this forces the Ω object diagonal conductivity as:


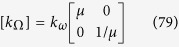


Note that, since we assumed μ = η to cancel the rotation, this conductivity matrix is now connected to the shape of the object via the μ coefficient. In the departure space, the conductivities are scalar and given by k_ω_ = 5 W·m^−1^·K^−1^ for the object to mimic and k_E\ω_ = k_0_ = 1 W·m^−1^·K^−1^ for its surrounding medium E\ω. Hence, the departure space is isotropic but non-homogeneous since k_ω_ ≠ k_E\ω_.

We considered a circular external frontier ∂E and 3 different shapes (see Fig. 3) for ∂ω: an ellipse (left figure), a flower (middle figure) and a butterfly (right figure). Their respective shape equations are as follows:


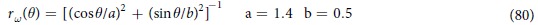



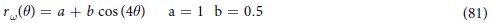



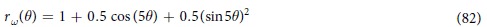


The last parameters to adjust in the H transformation are:

 r_0_ = 1.4 f_0_ = 0.7 for the ellipse case

 r_0_ = 1.5 f_0_ = 0.5 for the flower case

 r_0_ = 1.4 f_0_ = 0.7 for the butterfly case

From relations (73–77, 80–82), the cloaks were calculated following (8). The resulting conductivity matrices to perform the thermal illusion are given in Figs 4–6 in the polar basis. For each figure the 4 matrix parameters are given in color scales in the whole arrival space E, that is, for the Ω object and its cloak E\Ω. The colors can be saturated within Ω to emphasize the conductivity variations within the cloaks. In each figure, the matrix coefficients within Ω are constant, with k_rr_ = μ k_ω_, k_θθ_ = k_ω_/μ and k_rθ_ = k_θr_ = 0. In addition, there is an anisotropic gradient of conductivity within the cloak E\Ω.

Figure 4 is given for the ellipse of Fig. 3, with μ = 1 on the left and μ = 3 on the right. We notice in the right figure that the ellipse shape is lost; this results from the fact that, because of the r^μ^ power variation, the polar radius is increased or decreased, depending on whether r > 1 or r < 1. Figures 5 and 6 are given in a similar way for the 2 other shapes of Fig. 3 (flower and butterfly). Generally, we observe on a horizontal axis that, in the cloak E\Ω, the k_rr_(r,θ) behavior is connected to the ratio r_Ω_(θ)/r_ω_(θ). This is the manner by which the conductivity gradient compensates the ∂Ω shape to mimic ∂ω.

### Validation with heat flow pattern

Now that all cloaks have been designed, a final validation is required to calculate the resulting heat flow pattern in the two whole spaces (departure and arrival- see Fig. 1), that is:

 beyond the frontier ∂E limiting the ω object surrounded by E\ω in the departure space

 beyond the frontier ∂E limiting the Ω object surrounded with its cloak E\Ω in the arrival space

**Figure 7:**
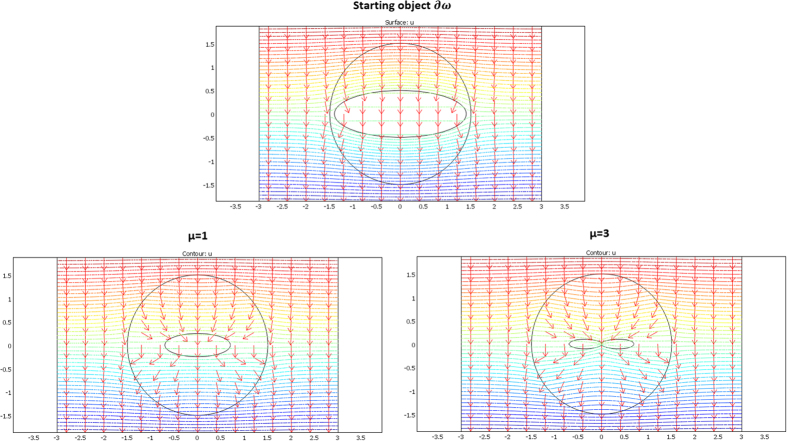
Case where ∂ω is an ellipse. Heat flow patterns are calculated with the conductivity matrices of Fig. 4. The top figure is for the scattering pattern of ω, and the bottom figures are for Ω surrounded by its cloak, with μ = 1 (left figure) and μ = 3 (right figure). All patterns are identical beyond the circular cloak ∂E.

**Figure 8:**
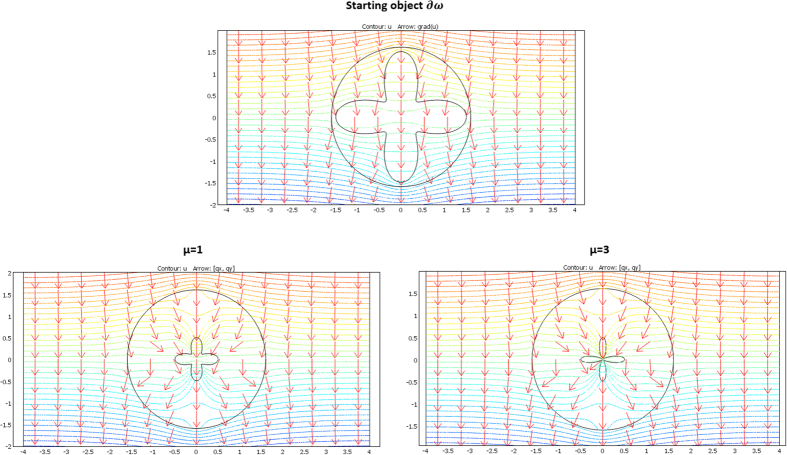
Case where ∂ω is a flower. Heat flow patterns are calculated with the conductivity matrices of Fig. 5. The top figure is for the scattering pattern of ω, and the bottom figures are for Ω surrounded by its cloak, with μ = 1 (left figure) and μ = 3 (right figure). All patterns are identical beyond the circular cloak ∂E.

**Figure 9:**
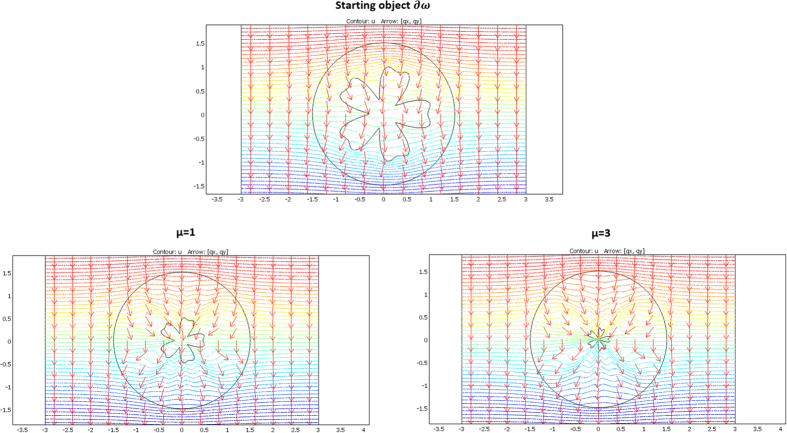
Case where ∂ω is a butterfly. Heat flow patterns are calculated with the conductivity matrices of Fig. 6. The top figure is for the scattering pattern of ω, and the bottom figures are for Ω surrounded by its cloak, with μ = 1 (left figure) and μ = 3 (right figure). All patterns are identical beyond the circular cloak ∂E.

The validation will be proven if we check that these fluxes beyond ∂E are identical for the two departure and arrival spaces; the results given in Fig 7, 8 and 9 were obtained with Comsol Multiphysics software. Because the thermal regime is static, no additional constraint originates from the ρC product. In all figures, the temperature is forced at the system entrance (top side of all drafts) and exit (bottom side of all drafts). All the previous conductivity matrices were implemented.

Figure 7 is given for the ellipse cases whose conductivity matrices are those of Fig. 4. The top figure is given in the departure space for the flux to mimic (that of ω), and the bottom figures are for the cloaked object (Ω) in the arrival space. The bottom left figure is calculated with μ = 1, and the right figure is for μ = 3. We observe for the 3 drafts that the heat flow pattern is invariant beyond the circular cloak, thereby validating all the results. In a similar manner, Figs 8 and 9 are given for the flower and the butterfly of Figs 5 and 6, respectively, providing successful results. All the results confirm that ω is indeed mimicked by Ω and its cloak.

### The case of rotation

**Figure 10:**
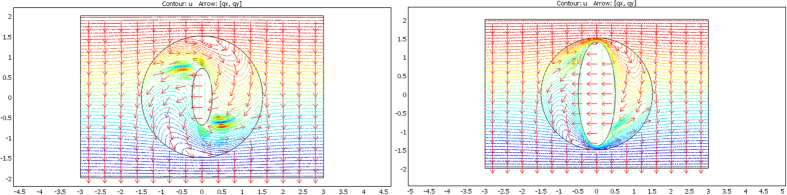
Flux pattern calculated for the horizontal ellipse of Fig. 7 after 90° rotation (right figure) completed by reduction (left figure). All fluxes are identical beyond the circular cloak.

**Figure 11:**
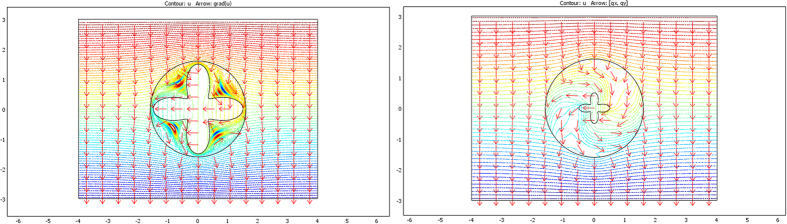
Flux pattern calculated for the flower of Fig. 8 after 90° rotation (left figure) completed by reduction (right figure). All fluxes are identical beyond the circular cloak.

**Figure 12:**
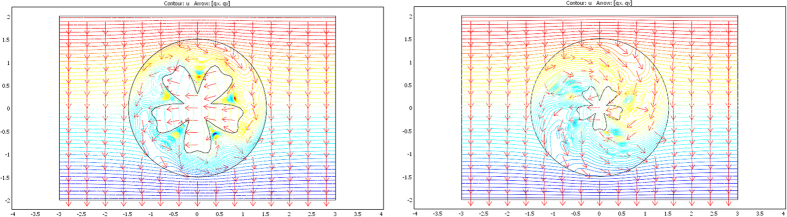
Flux pattern calculated for the butterfly of Fig. 9 after 90° rotation (left figure) completed by reduction (right figure). All fluxes are identical beyond the circular cloak.

**Figure 13:**
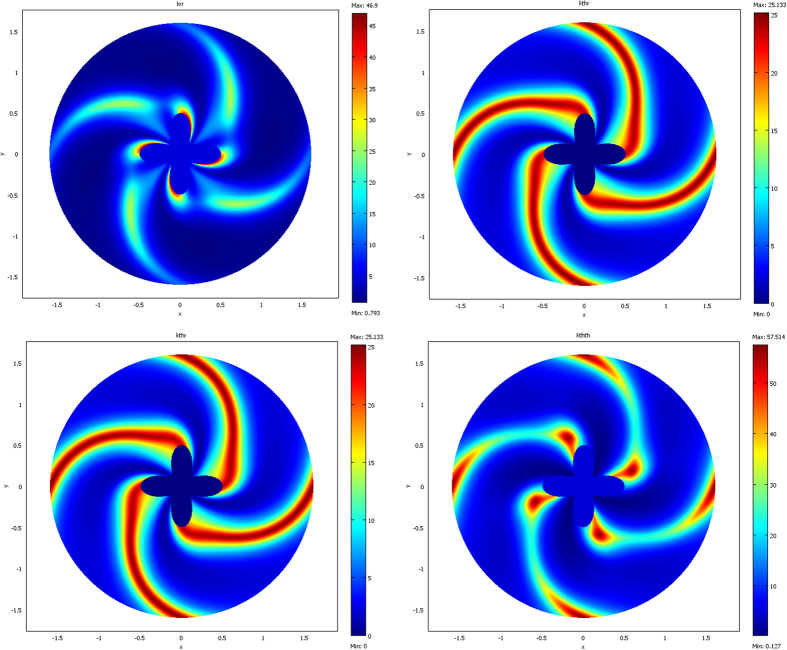
Conductivity matrix of the flower in the case of 90° rotation with no scaling.

Next, we address the validation in the case of rotation. Although the whole formulation is not modified when the objects are rotated (θ’≠θ), numerical implementation raises more difficulties. Indeed, additional intense polar gradients must be introduced (see eqs 73,74,75,76,77) that reduce the convergence speed in the heat equation and require mesh modification. However, all results are again successful, as shown in Fig 10, 11 and 12. In Fig. 10, the original object to mimic is the horizontal ellipse of Fig. 7; in the left of Fig. 10, the ellipse is rotated by 90° (θ_0_ = π/2), and it is both rotated and reduced in the right figure, with μ = 1, r_0_ = 1.4 and f_0_ = 0.7. We observe that, beyond the circular cloak, the heat flux patterns are not modified and remain identical to that of the top Fig. 7. Figures 11 and 12 are given for the flower and the butterfly, respectively with μ = 1, r_0_ = 1.5 and f_0_ = 0.5. The conclusions are the same. We notice for the flower that because it is invariant by 90° rotation, the mimicking process here occurs as if its anisotropic cloak were invisible. For completeness, we also show the conductivity matrix of the flower in Fig. 13.

### Dynamic regime

Throughout this paper, it was stressed that the additional ρC constraints (relation (7)) are not compatible with those of the conductivity (relation (6)) in the general case, which led us to reduce our analysis to the static regime. However, as stated above, there is one transformation that holds both the conductivity and the ρC product, and this transformation is characterized by a single rotation combined with a homothety (see relations (57–58)). These relations (57–58) are those that allow the mimicking technique in the transient regime for given isotropic objects, which we now validate with numerical calculation.

For the validation, the heat equation is solved in the dynamic regime. More exactly, we calculated the flux and temperature response of one non-zero Fourier component of the source (1 Hz) at time t = 0. The ρC product is 10 J⋅K^−1^ m^−3^ within the object ω to mimic, whereas it is 1 J⋅K^−1^ m^−3^ in its surrounding E\ω. The 3 shapes of the previous sections (ellipse, flower and butterfly) were considered with the same cloak conductivity matrices.

**Figure 14:**
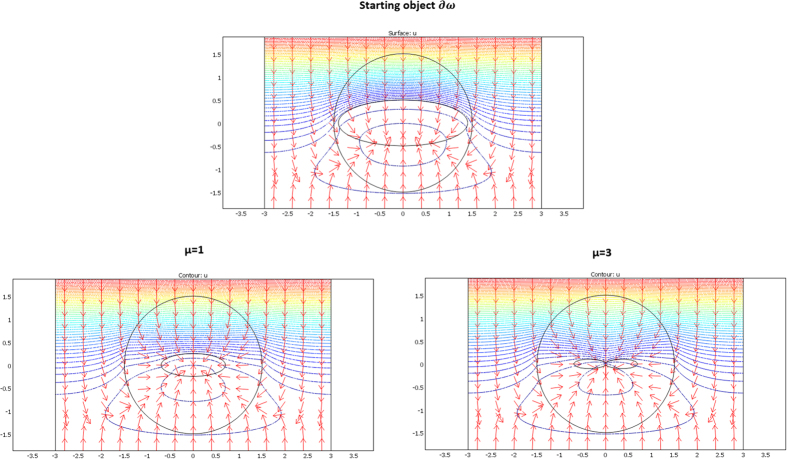
Legend analogous to Fig. 7, but for the dynamic regime.

**Figure 15:**
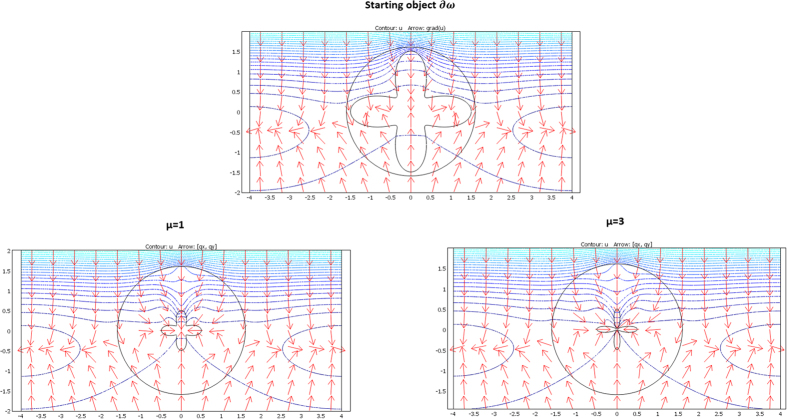
Legend analogous to Fig. 8, but for the dynamic regime.

**Figure 16:**
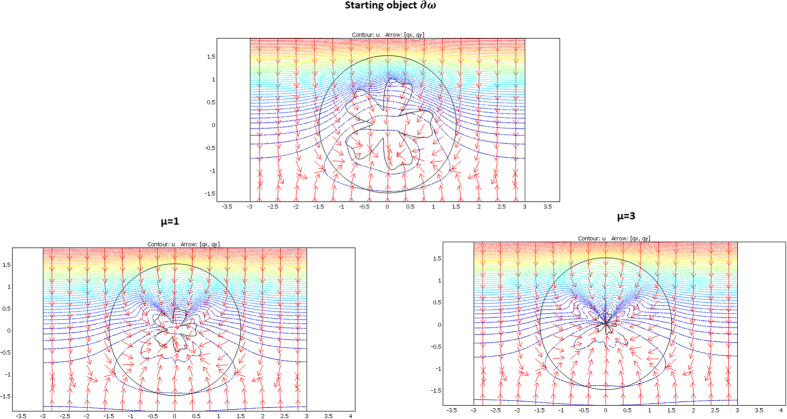
Legend analogous to Fig. 9, but for the dynamic regime.

The results shown in Fig 14, 15 and 16 confirm that successful data were obtained because all flux patterns are again found to be identical beyond the cloaks. Note that, in regard to the static regime, the heat fluxes above the cloak are far from those of the homogeneous case (scattering is stronger), so that the mimicking effect can be observed with more contrast. Validation of (57–58) is given by the bottom left figures that involve parameter (μ = 1), which holds both the conductivity and the ρC product. In the bottom right figures calculated with μ = 3, the ρC product was chosen to satisfy relation (7) and is therefore different from that of the object to mimic.

**Figure 17:**
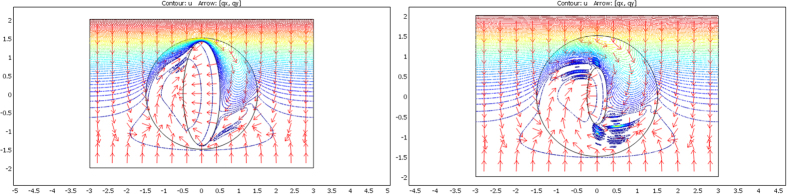
Legend analogous to Fig. 10, but for the dynamic regime. The left figure is for rotation, and the right figure is for rotation and scaling.

**Figure 18:**
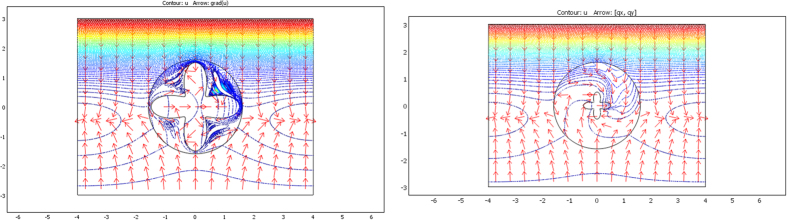
Legend analogous to Fig. 11, but for the dynamic regime. The left figure is for rotation, and the right figure is for rotation and scaling.

**Figure 19:**
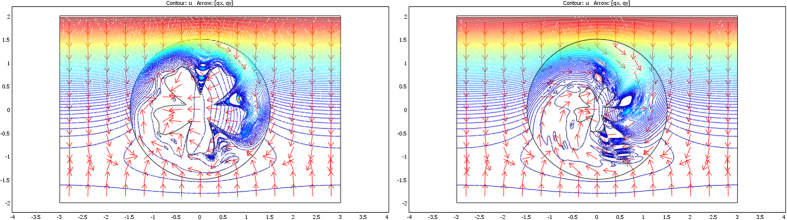
Legend analogous to Fig. 13, but for the dynamic regime. The left figure is for rotation, and the right figure is for rotation and scaling.

For completeness, we also plotted in Fig 17, 18 and 19 the harmonic flux patterns of the same objects after rotation (left figure) and scaling (right figure). As already noticed, the mimicking effect is easier to observe because of the strong scattering effects in the dynamic regime.

## Conclusion

We addressed a thermal inverse problem that consists of cloaking a predefined object (in shape and conductivity) to mimic the conduction heat flow of another object of given conductivity. Because all conductivities are given beforehand, the set of transformations that allow for passing from one object (to mimic) to another (to cloak) is reduced and was analytically and numerically calculated. This result allowed us to emphasize the class of shapes that can be mimicked when all conductivities are forced.

The solutions were first given for the heat flow in the spatiotemporal regime, on the basis of space transformation. To guarantee the existence of solutions, we first dropped a number of constraints related to the (ρC) products, leading us to the static regime with unique conditions on the conductivity matrices. The analytical calculation was fully developed in a 2D polar geometry for a heterogeneous isotropic object to mimic, and a heterogeneous diagonal object to cloak. Among all transformations, a sub-class was emphasized that allows for camouflaging of the diagonal objects. These transformations hold the conductivity and confer to the objects the adequate thermal illusion, that is, a heat flow pattern identical to that of another predefined object. It was also noticed how specific transformations allowed to match both the conductivity and the (ρc) constraints for the illusion to work in the dynamic regime.

Numerical calculation was performed for different object shapes to mimic an ellipse, a flower and a butterfly. Homothetic transformations were used for the cloaks at each polar angle, on the support of space continuity. For simplicity, we assumed that the ratio of the radial conductivity of the object to cloak to the scalar conductivity of the object to mimic was constant. Under these conditions, all mimicking predictions (including rotation and scaling) were confirmed with success, with a heat flow pattern identical for all objects above the cloaks. All these techniques can be directly extended to more general situations of asymmetric objects with arbitrary conductivities. We expect these results to bring added value for applications because they allow the camouflaging of objects that are chosen beforehand. In particular, they should make prototype design easier. In addition, the results open the door to camouflage in thermal radiation, at least for far-infrared wavelengths; indeed, at these wavelengths, where bulk absorption is dominant, the thermal radiation is mainly emitted from the surface of the external cloak, where the temperature has been shown to be invariant. Finally, generalization to other fields (such as waves) can also be directly performed.

Notice also that the most general case of a non-diagonal conductivity matrix that should be over-coated to mimic a non-isotropic matrix could be addressed in a similar manner; however, this would require full numerical calculation (including the inverse problem and additional partial differential equations).

## Additional Information

**How to cite this article**: Alwakil, A. *et al*. Inverse heat mimicking of given objects. *Sci. Rep.* **7**, 43288; doi: 10.1038/srep43288 (2017).

**Publisher's note:** Springer Nature remains neutral with regard to jurisdictional claims in published maps and institutional affiliations.
